# Effects of female preference intensity on the permissiveness of sexual trait polymorphisms

**DOI:** 10.1002/ece3.3957

**Published:** 2018-04-10

**Authors:** Aditya Ponkshe, John A. Endler

**Affiliations:** ^1^ Centre for Integrative Ecology School of Life & Environmental Sciences Deakin University Waurn Ponds Vic. Australia

**Keywords:** assortative mating, fisher process, null model, polymorphism permissiveness, sexual selection

## Abstract

Recent developments in sexual selection theory suggest that on their own, mate preferences can promote the maintenance of sexual trait diversity. However, how mate preferences constrain the permissiveness of sexual trait diversity in different environmental regimes remains an open question. Here, we examine how a range of mate choice parameters affect the permissiveness of sexual trait polymorphism under several selection regimes. We use the null model of sexual selection and show that environments with strong assortative mating significantly increase the permissiveness of sexual trait polymorphism. We show that for a given change in mate choice parameters, the permissiveness of polymorphism changes more in environments with strong natural selection on sexual traits than in environments with weak selection. Sets of nearly stable polymorphic populations with weak assortative mating are more likely to show accidental divergence in sexual traits than sets of populations with strong assortative mating. The permissiveness of sexual trait polymorphism critically depends upon particular combinations of natural selection and mate choice parameters.

## 1 INTRODUCTION

Substantial variation is recorded in sexually selected traits within species (Brooks & Endler, 2001; Gray & McKinnon, 2007; Pomiankowski & Møller, 1995; Wellenreuther, Svensson, & Hansson, 2014). Sexual selection by female choice can contribute to the maintenance of sexual trait polymorphism (Brooks, 2002; Chunco, McKinnon, & Servedio, 2007; Gray & McKinnon, 2007; Hughes, Houde, Price, & Rodd, 2013; Kingston, Rosenthal, & Ryan, 2003; M'Gonigle, Mazzucco, Otto, & Dieckmann, 2012; Wellenreuther et al., 2014). However, we know little about how female choice affects the permissiveness of sexual trait polymorphism.

The Fisher process is an engine of trait‐preference coevolution and remains at the heart of sexual selection, in fact, it could be called the sexual selection null hypothesis (Prum, 2010). Genetic models involving the Fisher process normally identify equilibrium values and stability of male ornaments–female preferences and examine which conditions promote or constrain the trait‐preference exaggeration (Mead & Arnold, 2004) and speciation (See Gavrilets, 2004; Higashi, Takimoto, & Yamamura, 1999; Kirkpatrick & Ravigné, 2002; Servedio & Bürger, 2014 for detailed discussions).

The Fisher process of sexual selection produces a stable line of equilibria in addition to Fisherian runaway (Kirkpatrick, 1982; Kirkpatrick & Ryan, 1991; Lande, 1981). The stable line of equilibria is normally interpreted as evidence for quantitative variation in both sexual traits and mate preferences among populations. Populations can move along the equilibrium line by random drift and can diverge and speciate due to differences in equilibrium values (Houde, 1993; Kirkpatrick, 1982; Prum, 2010; Uyeda, Arnold, Hohenlohe, & Mead, 2009). Additional selection on sexual traits and/or on mate preferences (selection independent of mate choice) affects the stability of equilibrium line. Consequently, any changes in the shape of equilibrium line imply meaning, honesty, and design in intersexual signals (Fuller, Houle, & Travis, 2005; Prum, 2010). Direct selection on sexual traits only changes the slope of equilibrium line. However, the stable line of equilibria disappears and collapses to a single equilibrium point when mate preferences are under even weak directional selection (Grafen, 1990; Kirkpatrick & Ryan, 1991; Prum, 2010). Although direct selection on mate preferences drastically alters the stability of the equilibrium line, the Fisherian runaway process can still continue even when mate preferences are under strong selection (Hall, Kirkpatrick, & West, 2000).

Kirkpatrick's haploid version of the null model of sexual selection (Kirkpatrick, 1982) and subsequent models expanding Kirkpatrick's original model (Bulmer, 1989; Seger, 1985; Seger & Trivers, 1986; Takahasi, 1997) analyze the effects of mate preference strengths on the shape and stability of the equilibrium line. In contrast, here, we investigate what can happen away from the line. In this paper, we use Kirkpatrick's model and focus on how mate preferences affect the size and shape of the attraction basin around the stable line of equilibria. We use the dynamical systems theory approach (Beisner, Haydon, & Cuddington, 2003; Meyer, 2015) and use the size and shape of attraction basins as measures of permissiveness of polymorphism as a way to estimate the possible effects of random perturbations of populations away from the line. An attraction basin is the set of all starting male–female allele frequency combinations from which populations in a given environment evolve to a set of polymorphic equilibria within the basin. The size and shape of an attraction basin is a measure of the permissiveness of sexual trait polymorphism in a given set of environmental conditions; the larger the basin, it is the less likely a given perturbation will draw a population outside the zone of attraction and lose its polymorphism. Permissiveness of sexual trait polymorphism can be defined as the capacity of the system to allow the polymorphism to be maintained in potentially variable conditions.

We examine how mate choice parameters, other selective forces independent of mate choice (natural selection), and their interactions, affect the permissiveness of sexual trait polymorphism. It is important to note that diploid models involving the Fisher process can show differences in evolutionary dynamics compared with the haploid version (Greenspoon & Otto, 2009). We use Kirkpatrick's haploid version of model and examine two cases for the permissiveness of sexual trait polymorphism. In the first set of models, the sexual trait is only affected by selective mating, whereas, in the second, directional viability selection also affects the sexual trait.

## 2 MODELS AND RESULTS

### 2.1 Model 1: Mate choice only (true null model of intersexual selection)

We use the classic haploid version of the null model of sexual selection developed by Kirkpatrick as a foundation. Kirkpatrick's model assumes directional viability selection on male traits in addition to mate choice. Here, we first work with a null model with no viability selection. The only difference between our model 1 and Kirkpatrick's (Kirkpatrick, 1982) second model is that our model does not include viability selection on sexual traits, whereas Kirkpatrick's model does include it. The literature on sexual selection utilizes three important preference functions: fixed relative preference, best of *N* males, and absolute preference function. Kirkpatrick's original models use the fixed relative preference function. Here, we also use the fixed relative preference framework used by Gavrilets (Gavrilets, 2004) which has a slightly different way of parametrization than the Kirkpatrick's original model. Apart from these modifications, our basic models (model 1 and 2) are identical with Kirkpatrick's model (Kirkpatrick, 1982). Previous work has concentrated on the structure and stability of the line of equilibrium. Here, we explore the effects of the size and shape of the zone of attraction around the equilibrium line in order to assess effects of random fluctuations away from the line.

Consider a haploid population exhibiting polymorphism in both sexual traits and in mating preferences for the sexual traits. Assume locus T controls male traits and an unlinked locus P controls female preference for the male traits. Let each locus have two alleles which correspond to different phenotypes, T_1_, T_2_ for different sexual traits in males and P_1_, P_2_ for different female preferences. Let *m*_1_, *m*_2_, *m*_3,_ and *m*_4_ be the frequencies of T_1_P_1_, T_1_P_2_, T_2_P_1,_ and T_2_P_2,_ respectively, in males and *f*_1_, *f*_2_, *f*_3,_ and *f*_4_ in females. For every combination of starting frequencies and zygote frequencies, *m*_i_ = *f*_j_ and *∑m*_i_ = *∑ f*_j_ = 1. Note that there is no cost associated with mate preferences and sexual traits.

Let the relative preference of a P_1_ female for T_1_ males be 1 and her preference for T_2_ males be 1‐α_1_. Similarly, let the preference of P_2_ females for T_2_ males be 1 and her preference for T_1_ males be 1‐α_2_ *,* where α_1_ and α_2_ are mate choice parameters (discrimination coefficients) measuring the strength of preference. If α_1_ = α_2_ = 0, there is no choice with respect to male traits and α_1_ = α_2_ = 1 means both females only chose their preferred traits (complete positive assortative mating). Recurrence equations for zygote frequencies in the next generation are

(1a) $$\begin{array}{cc} T_{1}{P_{1}}_{\left(t+1\right)}= & \;f_{1}\left(\frac{m_{1}}{z_{1}}+\frac{m_{2}}{2z_{1}}+\frac{m_{3}\;\left(1-\alpha_{1}\;\right)}{2z_{1}}+\frac{m_{4}\;\left(1-\alpha_{2}\;\right)}{4z_{1}}\right)\\ & +\;f_{3}\left(\frac{m_{1}}{2z_{1}}\;+\frac{m_{2}}{4z_{1}}\right)+f_{2}\left(\frac{m_{1}\;\left(1-\alpha_{2}\;\right)}{2z_{2}}+\frac{m_{3}}{4z_{2}}\right)\\ & +\;f_{4}\left(\frac{m_{1}\left(1-\alpha_{2}\;\right)}{4z_{2}}\right) \end{array}$$

(1b) $$\begin{array}{cc} T_{1}{P_{2}}_{\left(t+1\right)}= & \;f_{2}\left(\frac{m_{1}\left(1-\alpha_{2}\right)}{2z_{2}}+\frac{m_{2}\left(1-\alpha_{2}\;\right)}{z_{2}}+\frac{m_{3}}{4z_{2}}+\frac{m_{4}}{2z_{2}}\right)\\ & +\;f_{4}\left(\frac{m_{1}\left(1-\alpha_{2}\;\right)}{4z_{2}}+\frac{m_{2}\left(1-\alpha_{2}\;\right)}{2z_{2}}\right)+\;f_{1}\left(\frac{m_{2}}{2z_{1}}+\frac{m_{4}\left(1-\alpha_{1}\;\right)}{4z_{1}}\right)\\ & +\;f_{3}\left(\frac{m_{2}}{4z_{1}}\right) \end{array}$$

(1c) $$\begin{array}{cc} T_{2}{P_{1}}_{\left(t+1\right)}= & \;\;f_{1}\left(\frac{m_{3}\left(1-\alpha_{1}\right)}{2z_{1}}+\frac{m_{4}\left(1-\alpha_{1}\right)}{4z_{1}}\right)\\ & +\;f_{3}\left(\frac{m_{1}}{2z_{1}}+\frac{m_{2}}{4z_{1}}+\frac{m_{3}\left(1-\alpha_{1}\right)}{z_{1}}+\frac{m_{4}\left(1-\alpha_{1}\right)}{2z_{1}}\right)\\ & \;+\;f_{2}\left(\frac{m_{3}}{4z_{2}}\right)+f_{4}\left(\frac{m_{1}\left(1-\alpha_{2}\right)}{4z_{2}}+\frac{m_{3}}{2z_{2}}\right) \end{array}$$

(1d) $$\begin{array}{cc} T_{2}{P_{2}}_{\left(t+1\right)}= & \;f_{2}\left(\frac{m_{3}}{4z_{2}}+\frac{m_{4}}{2z_{2}}\right)\\ & \;+\;f_{4}\left(\frac{m_{1}\left(1-\alpha_{2}\right)}{4z_{2}}+\frac{m_{2}\left(1-\alpha_{2}\right)}{2z_{2}}+\frac{m_{3}}{2z_{2}}+\frac{m_{4}}{z_{2}}\right)\\ & \;+\;f_{1}\left(\frac{m_{4}\left(1-\alpha_{1}\right)}{4z_{1}}\right)+f_{3}\left(\frac{m_{2}}{4z_{1}}+\frac{m_{4}\left(1-\alpha_{1}\right)}{2z_{1}}\right) \end{array}$$

Here,

$$\;z_{1}=m_{1}+m_{2}+\left(1-\alpha_{1}\right)\left(m_{3}+m_{4}\right);\;z_{2}=\left(1-\alpha_{2}\right)\left(m_{1}+m_{2}\right)+m_{3}+m_{4}$$

T_1_ frequencies were computed numerically by iterating the equations for 30,000 generations for all combinations of male–female starting frequencies and α_1_ and α_2_ using MATLAB 2015b. We found 30,000 generations to be more than sufficient time for the populations to attain a stable equilibrium for the entire range of α_1_ and α_2_.

**Figure 1:**
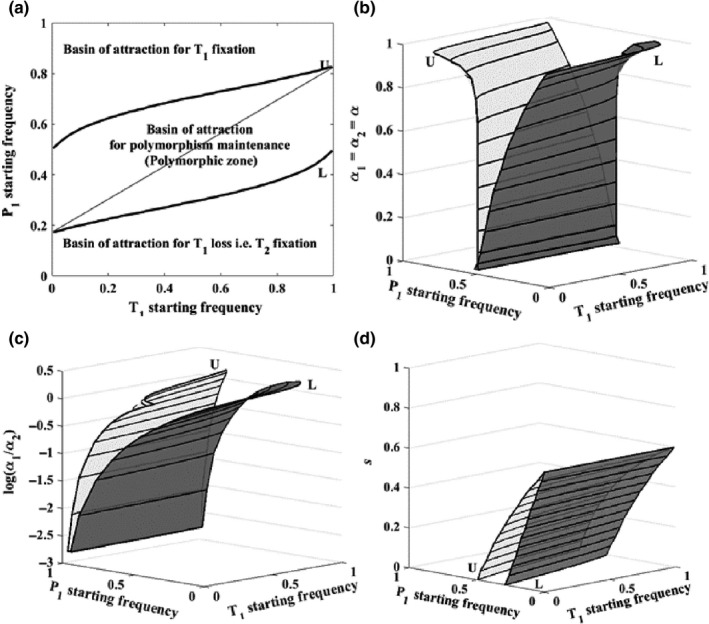
Effects of selection parameters (α_1_ *,* α_2_ and *s*) on polymorphic zones. (a) Phase map showing attraction basin of polymorphic equilibria (polymorphic zone), delimited by two thresholds, U and L (thick black curves) for α_1_ = α_2_ = α = 0.8. The thin black line is the theoretical stable line of polymorphic equilibria. (b) Changes in the polymorphic zone when mate preferences are equal in both female types (α = α_2_ = α*),* varying α. (c) Changes in the polymorphic zone for unequal mating preferences: varying α_1_ and holding α_2_ constant and strong (α_2_ = 0.8). (d) Changes in the polymorphic zone as a function of viability selection strength *s* for α_1_ = α_2_ = 0.6. Each combination of α_1_ and α_2_ or *s* (vertical axis) in b, c, and d correspond with one upper and one lower boundary and forms one polymorphic zone. Dark black lines on the light gray surface (U) are upper boundaries and those on the dark gray surface (L) are lower boundaries. Note the differences in shape and size of polymorphic zones. Starting frequencies of P_1_ and T_1_ alleles anywhere inside U and L boundaries maintain polymorphism in T in the future. Starting points outside U and L surfaces lose T polymorphism in the future

For a given constant α_1_ and α_2_, joint initial male–female allele frequencies that will maintain sexual trait polymorphism at equilibrium form a zone in the joint frequency state space with two distinct boundaries (Figure 1a). We will refer to the central zone as the polymorphic zone and boundaries as the upper (U) and lower (L) boundaries by where they intersect the axis of P_1_ starting frequencies (Figure 1a).

To determine the polymorphic zone and boundaries, we first computed the equilibrium T_1_ frequency (T_1_ frequency after 30,000 generations) for all possible combinations of starting frequencies of T_1_ and P_1_ for a given constant α_1_ and α_2_. The polymorphic zone is an attraction basin for polymorphic equilibria; it represents all joint T_1_ and P_1_ starting frequencies that eventually produce the equilibrium T_1_ frequency between 0.001 and 0.999 (0.001 ≤ T_1 (equilibrium frequency)_ ≤0.999). To compute U (the boundary separating the polymorphic zone and the attraction basin for T_1_ fixation), we identified unique threshold starting frequencies of P_1_ for the entire range of T_1_ starting frequencies such that any change in starting frequency of P_1_ above the threshold will result in T_1_ fixation, that is, T_1(equilibrium frequency)_ >0.999. Similarly, to compute L (the boundary separating the polymorphic zone and the attraction basin for T_2_ fixation), we identified the threshold starting frequencies of P_1_ such that any change in the starting frequency of P_1_ below this threshold will result in T_1_ loss, that is, T_1(equilibrium frequency)_ <0.001.

U and L separate very different evolutionary outcomes. Populations with joint male–female allele frequencies starting anywhere inside the central zone (within U and L) retain sexual trait polymorphisms. Populations with joint frequencies starting anywhere outside the central zone lose sexual trait polymorphism (either T_1_ is fixed or it is lost).

The area of the attraction basin is a measure of the permissiveness of sexual trait polymorphism in a given set of environmental conditions. If the area of the polymorphic zone is small, then the permissiveness is low and if the area is large, then the permissiveness is high. If random factors change allele frequencies, then low permissiveness implies a greater chance that a changed allele frequency combination will cross a boundary (leading to loss or fixation) than the same change under conditions of high permissiveness. In addition, changes in mate choice parameters (α_1_ and α_2_) alter the size and shape of the polymorphic zone, affecting the permissiveness (Figure 1). We explored the effects of α_1_ and α_2_ on the area of the polymorphic zone.

#### 2.1.1 Effects of choice parameters on the permissiveness of sexual trait polymorphism

Different combinations of α_1_ and α_2_ alter the position, shape, and size of the polymorphic zone (Figures 1b and c). When preferences are nearly equal and weak, the polymorphic zone remains narrow; the system has low permissiveness. As α_1_ = α_2_ = α increases, the zone boundaries (U and L) gradually move apart and the polymorphic zone becomes broad (note the gradual increase in the area of polymorphic zone with α in Figure 1b). In a broad zone, polymorphic populations near the equilibrium line are much less likely to be sensitive to perturbations in male–female allele frequencies than narrow zones because the zone boundaries are less likely to be crossed by a temporary change in gene frequencies. Gene frequency perturbations could occur either as a result of genetic drift or changing environments but the effect on the polymorphism will be the same.

**Figure 2:**
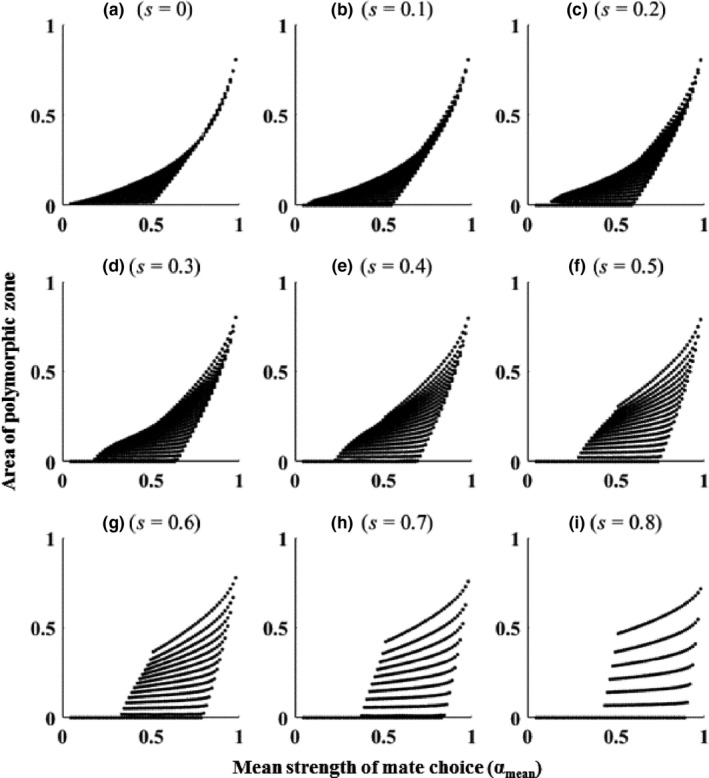
Relationship between the area of the polymorphic zone (permissiveness of sexual trait polymorphism) and the mean strength of mate choice (α_mean_) under different viability selection (*s*) regimes Each dot in the panels a–i represents the area of the polymorphic zone for a unique combination of α_1_ and α_2_ across the entire range of possible values of α_1_ and α_2_

Different combinations of α_1_ and α_2_ alter the polymorphic zone area in different ways. Figure 2 shows the relationship between the area of the polymorphic zone (permissiveness of sexual trait polymorphism) and the mean strength of mate choice (α_mean_) under different viability selection (*s*) regimes over the entire range of possible values of α_1_ and α_2_. Figure 2a shows the relationship between the polymorphic zone area (the permissiveness of sexual trait polymorphism) and the mean strength of mate choice (α_mean_) in the absence of any other selection apart from mate choice. The rate of change of permissiveness as a function of α_1_ and α_2_ is smaller when α_1_ and α_2_ are weak and it increases as α_1_ and α_2_ become stronger.

Permissiveness of polymorphism (polymorphic zone area) increases disproportionately as a function of the mean strength of mate choice (α_mean_) (Figure 2a). There are unique maximum and minimum values of permissiveness for a constant α_mean_ (Figure 2a). The maximum and minimum values of permissiveness are biologically important because they indicate the uppermost and lowermost limits of permissiveness that populations can achieve in the given environment. For a given mean strength of mate choice, permissiveness cannot exceed the maximum value of permissiveness and/or cannot go below the minimum value of permissiveness. Permissiveness can vary within these limits depending on the relative and absolute values of α_1_ and α_2_. The maximum and minimum values remain very low (close to zero) when α_mean_ is weak. Maximum and minimum permissiveness values increase disproportionately as α_mean_ increases and both remain high for stronger α_mean._ Differences in α_1_ and/or α_2_ among populations can cause disproportionately large differences in the permissiveness of sexual trait polymorphisms; even if the populations are identical in their α_mean_.

### 2.2 Model 2: Sexual Trait (T) under directional viability selection

The standard model of intersexual selection (Kirkpatrick, 1982; Prum, 2010) normally assumes directional viability selection on male traits in addition to mate choice. For example, viability selection could be caused by the physical environment. Our model 2 is same as Kirkpatrick's second model in (Kirkpatrick, 1982). Here, we explored the range of permissiveness when male traits are under directional viability selection independent of the preference trait P.

Let T_2_ males have a disadvantage such that the T_2_ trait viability is 1‐*s* relative to T_1_ males; *s* is the viability selection coefficient (0 ≤ *s *≤* *1). Let viability selection on males occur before selective mating; this alters the frequencies of males available for mating. Aside from this modification, the model is the same as Model 1 (a true null model without viability selection on male traits). New gamete frequencies available for mating in males are $m_{1}^{\prime }=\frac{m_{1}}{\overset{¯}{W}};m_{2}^{\prime }=\frac{m_{2}}{\overset{¯}{W}};m_{3}^{\prime }=\frac{m_{3}\left(1-s\right)}{\overset{¯}{W}};m_{4}^{\prime }=\frac{m_{4}\left(1-s\right)}{\overset{¯}{W}};\;\overset{¯}{W}=1-st_{2}\;\text{where}\;t_{2}=m_{3}+m_{4}$ Zygote frequencies in the next generation can be obtained by substituting $m_{1}^{\prime }$ *,* $m_{2}^{\prime }$ *,* $m_{3}^{\prime }$ *,* and $m_{4}^{\prime }$ for *m*_1_, *m*_2_, *m*_3,_ and *m*_4_ in Equations 1a to 1d. Note that *m*_1_, *m*_2_, *m*_3,_ and *m*_4_ are the frequencies of T_1_P_1_, T_1_P_2_, T_2_P_1,_ and T_2_P_2,_ respectively, in males.

T_1_ frequencies were computed numerically by iterating Equations 1a to 1d after substituting new gamete frequencies for all combinations of male–female starting frequencies and α_1_ and α_2_ using MATLAB 2015b. Five thousand generations were more than sufficient for populations to reach a stable equilibrium.

#### 2.2.1 Joint effects of mate choice and natural selection parameters on the permissiveness of sexual trait polymorphism

The strengths of *s*, α_1,_ and α_2_ have interacting effects and this determines the size, shape, and position of the polymorphic zone (Figure 1d shows changes in the zone as a function of *s* for α_1_ = α_2_ = 0.6). Figure 2 shows how the permissiveness changes as a function of α_mean_ under different viability selection regimes.

For a constant α_mean_, *s* increases the difference between the maximum and minimum values of permissiveness and thus, effectively increases the permissiveness range. This suggests that for the same change in α_1_ and/or α_2_, the permissiveness of polymorphism changes more in environments with strong *s* than weak *s* (each dot in Figure 2a–i represents the area of the polymorphic zone for the unique combination of α_1_ and α_2_).

## 3 DISCUSSION

Classical theory suggests that on its own, selective mating should reduce the variance in sexually selected traits (Kirkpatrick & Ryan, 1991; Pomiankowski & Møller, 1995), yet many species show variation in these traits (Brooks & Endler, 2001; Gray & McKinnon, 2007; Pomiankowski & Møller, 1995; Wellenreuther et al., 2014). Recent developments in sexual selection theory suggest that on their own, mate preferences can promote the maintenance of sexual trait diversity and promote coexistence (M'Gonigle et al., 2012). However, how mate preferences constrain the maintenance of sexual trait diversity in different environmental regimes remains an open question. Our study shows that the permissiveness of sexual trait polymorphism increases in environments with strong selective mating; the risk of loss of sexual trait diversity is significantly lower when preferential mating is strong compared to when it is weak. Now we discuss a potential mechanism which can produce these results.

Selective mating produces an overall negative frequency‐dependent effect which makes the line of polymorphic equilibria a stable attractor (Seger, 1985). For a constant frequency of the preference allele in populations, male traits exhibit higher fitness relative to the other trait when lower in frequency (see figure 1b in Seger, 1985). Thus, populations starting with a higher frequency of male alleles require a higher threshold frequency of corresponding female alleles to continue the Fisher process and lead populations to fixation. This is the reason that U and L in Figure 1a are curved and not horizontal straight lines. Strong assortative mating amplifies the negative frequency‐dependent effect; note how Figure 1b shows that U and L remain straight horizontal lines when α is weak but become more curved as α becomes strong. This can potentially increase the size of attraction basin and make polymorphic attractors (stable line of polymorphic equilibria) significantly more robust to random factors when assortative mating is strong than when it is weak.

These results have strong implications for accidental population divergence because populations which move both above the upper (U) and below the lower (L) polymorphic boundaries will result in fixation of different sexual trait alleles. Note that populations diverge but remain polymorphic if they stay inside the polymorphic zone. Populations fix the same trait if they fall on the same side of the zone. Two populations show divergent fixation (fixation of different traits) if they fall on the opposite sides of the polymorphic zone. Note that we are only considering populations which are polymorphic for both P and T because if one is fixed, then divergence is unlikely until a mutation makes it polymorphic again.

Selective conditions that enhance the permissiveness reduce the potential for accidental divergent fixation or loss of allele among polymorphic populations that sit on or near the line of polymorphic equilibria, compared to conditions that reduce the permissiveness of polymorphisms. When the assortative mating is strong, then the polymorphic zone remains broad (greater permissiveness) and hence a relatively larger perturbation in male–female gene frequencies is required to cause accidental divergence in sexual traits among isolated polymorphic populations that sit on the line of equilibria (i.e., to throw populations across the zone boundaries in opposite directions). Thus, for the same magnitude of large gene frequency fluctuations, accidental population divergence is less likely in conditions that enhance the permissiveness such as strong assortative mating. In contrast, when the assortative mating is weak then the polymorphic zone remains narrow and relatively small perturbations in allele frequencies can easily push populations beyond the polymorphic zone boundaries (lower permissiveness). Consequently, our results suggest that for the same range of male–female gene frequency perturbations, sets of isolated and nearly stable polymorphic populations (populations sitting at different positions on or near the line of equilibria in Figure 1a) with strong (but not complete) assortative mating are less likely to show accidental divergence than sets of populations with weaker assortative mating. In other words, sets of isolated polymorphic populations near the line of equilibria are less likely to cross the zone boundaries in opposite directions when assortative mating is strong than when it is weak.

The Fisher process on its own reduces the likelihood of sexual trait divergence in the face of gene flow between populations with different natural selection parameters (Servedio, 2016; Servedio & Bürger, 2014). Our results suggest that in the absence of gene flow, for a given viability selection regime, strong mate preferences reduce the potential of accidental divergence among isolated polymorphic populations which sit near the stable line of polymorphic equilibria. Sets of isolated polymorphic populations near the line of equilibria are less likely to cross the zone boundaries in opposite directions and are less likely to diverge accidentally when assortative mating is strong than when it is weak. The relationship between gene flow and the permissiveness of sexual trait polymorphisms is entirely unexplored. For example, there is a possibility that a combination of isolation‐by‐distance gene flow and very strong preferences may permit some polymorphisms within populations. One interesting possibility is very low gene flow where the alleles coming into the population of interest fluctuate at random such that directional bias changes in time.

Gavrilets (Gavrilets, 2004) used a hybrid deficiency index (*I*) to measure the potential for reproductive isolation in a model identical to our Model 1. He found that hybrids are maintained in populations even if both females show strong mating preferences. Hybrids are eliminated only when mating preferences are extremely strong (but not completely assortative). For example when α_1_ = α_2_ = α > 0.9, see figure 9.5 in Gavrilets (2004). Our results show that strong mating preferences make sexual trait polymorphisms more permissive. Thus, for strong α, polymorphic populations sitting on or close to the stable line may not necessarily develop reproductive isolation and can remain polymorphic for long periods.

In summary, strong assortative mating significantly increases the permissiveness of sexual trait polymorphism under a broad range of environmental regimes. These results suggest that early stages of population divergence by accident could stall especially with strong mating preferences, and further parametric changes may need to occur before complete divergence.

## CONFLICT OF INTEREST

None declared.

## AUTHOR CONTRIBUTIONS

Models were designed and analyzed by AP. The manuscript was prepared by AP and was revised by JAE.
